# VOTER OPINIONS ON BRAIN HEALTH AND EARLY DETECTION OF DEMENTIA IN LOS ANGELES COUNTY

**DOI:** 10.1093/geroni/igae098.2308

**Published:** 2024-12-31

**Authors:** Noel Barragan, Mariana Reyes, Mark DiCamillo, Tony Kuo

**Affiliations:** Los Angeles County Department of Public Health, Los Angeles, California, United States; Los Angeles County Department of Public Health, Los Angeles, California, United States; University of California Berkeley, Berkeley, California, United States; Los Angeles County Department of Public Health, Los Angeles, California, United States

## Abstract

The burden of dementia continues to expand as the population ages. To address this growing public health problem, the local health department in Los Angeles County (LAC) launched its Healthy Brain LA initiative to encourage individuals and communities to prioritize brain health as a strategy for reducing the risk and impact of dementia. To better understand local perceptions about dementia and to inform the initiative’s efforts, public health partnered with the Berkely IGS Poll to survey LAC voters on key topics related to brain health. The online poll was administered in January 2024 via email invitations sent out to stratified random samples of registered voters in the county; 1,775 respondents competed the survey. Of the total sample, 16% indicated they currently help with care for an adult with memory problems. Overall, 70% reported being somewhat or very worried about the possibility of developing dementia. A majority (84%) agreed they could take steps to improve their brain health and reduce their risk of dementia. Notably, only 13% reported having ever discussed ways to prevent dementia with their doctor. Nearly two-thirds (62%) strongly agreed that they would want to receive a dementia diagnosis as early as possible. There is increasing value in approaching dementia through a public health prevention lens, emphasizing risk reduction and early detection as strategies for addressing this condition. Poll results offer important insights into potential ways in which public health can help activate public engagement and health system involvement in dementia prevention, care and support.

